# Synthesis and Characterization of Activated Biocarbons Produced from Avocado Seeds Using the Non-Toxic and Environmentally Friendly Activating Agent K_2_CO_3_ for CO_2_ Capture

**DOI:** 10.3390/molecules30234658

**Published:** 2025-12-04

**Authors:** Joanna Siemak, Beata Michalkiewicz

**Affiliations:** Department of Catalytic and Sorbent Materials Engineering, Faculty of Chemical Technology and Engineering, West Pomeranian University of Technology in Szczecin, Piastów Ave. 42, 71-065 Szczecin, Poland; joanna.siemak@zut.edu.pl

**Keywords:** activated biocarbon, avocado seeds, CO_2_ capture, K_2_CO_3_

## Abstract

Activated biocarbons were synthesized from avocado seeds using potassium carbonate as an activating agent. The study aimed to evaluate K_2_CO_3_ as a greener and less corrosive alternative to KOH, traditionally used for producing porous carbons. Twelve samples were obtained under varying activation conditions using both dry K_2_CO_3_ and its saturated solution. The material activated at 800 °C with a 1:1 precursor-to-activator ratio (C_K_2_CO_3__800) showed the highest CO_2_ adsorption capacity of 6.26 mmol/g at 0 °C and 1 bar. Nitrogen adsorption–desorption analysis confirmed a predominantly microporous structure, with ultramicropores (0.3–0.7 nm) primarily responsible for the high CO_2_ uptake. The Sips model provided the best fit to the adsorption equilibrium data, indicating a heterogeneous surface. The isosteric heat of adsorption (22–26 kJ/mol) confirmed a physical adsorption mechanism. Furthermore, the CO_2_/N_2_ selectivity, evaluated using the Ideal Adsorbed Solution Theory (IAST), reached values up to 18 at low pressures, highlighting the excellent separation performance. These findings demonstrate that avocado seed-derived activated carbons prepared with K_2_CO_3_ are efficient, renewable, and environmentally friendly sorbents for CO_2_ capture, combining high adsorption capacity with sustainability and ease of synthesis.

## 1. Introduction

Carbon dioxide (CO_2_) released into the atmosphere as a result of anthropogenic activities, such as the combustion of fossil fuels, industrial processes, and deforestation, is widely recognized as the primary driver of global warming and climate change. The continuously increasing atmospheric concentration of CO_2_ enhances the greenhouse effect by trapping outgoing infrared radiation, which leads to a progressive rise in global mean temperatures, disturbance of climatic patterns, and severe environmental consequences [1].

To mitigate these adverse effects, a wide range of strategies have been developed to limit CO_2_ emissions or remove it from the atmosphere. Among them, carbon capture and sequestration (CCS) has emerged as one of the most promising approaches. This strategy involves capturing CO_2_ from large point sources, such as power plants and industrial facilities, followed by its transportation and long-term storage in geological formations or its utilization in chemical, biological, or mineralization processes [2].

Adsorption is one of the most effective and widely applied techniques for gas separation due to its simplicity, high efficiency, and low energy consumption compared with other methods. Unlike absorption, membrane separation, or cryogenic distillation, adsorption processes can operate efficiently at relatively low temperatures and pressures. Moreover, they allow for the selective removal or recovery of specific gas components, such as CO_2_, using solid porous materials with tailored surface properties. Hence, designing adsorbent materials that exhibit superior affinity and capacity for the target gas is essential to ensure effective adsorption performance [3].

Among various materials investigated for this purpose, activated carbons have attracted significant attention due to their well-developed porous structure, large specific surface area, chemical stability, and tunable surface functionality [4,5]. These features make them particularly suitable for CO_2_ capture, as they enable high adsorption capacities under mild operating conditions and excellent regenerability over multiple cycles. Furthermore, activated carbons can be synthesized from a wide range of low-cost and renewable precursors, including agricultural and industrial wastes, which enhances their environmental and economic attractiveness [6,7].

Activated carbons can be synthesized from a wide variety of precursors using different preparation techniques. Among these methods, chemical activation is of particular importance and is the most commonly employed approach for producing materials with a well-developed pore structure [8,9]. In this process, various chemical agents are used as activating substances, including KOH [10,11,12], ZnCl_2_ [13], H_3_PO_4_ [14], and NaOH [15]. Among these activating agents, KOH is the most widely used, as it enables the formation of materials with a high specific surface area and a well-developed hierarchical porosity [16,17].

The use of KOH as an activating agent in the production of activated carbons involves several significant drawbacks that limit its large-scale application [17,18,19]. Activation with KOH generates large amounts of wastewater containing dissolved potassium salts, mainly K_2_CO_3_, K_2_O, and K_2_SiO_3_. These wastes require neutralization and disposal, which substantially increases both the operational costs and the environmental impact of the process. Another disadvantage is the strong corrosive nature of KOH, which necessitates the use of chemically resistant reactors, pipelines, valves, and specialized laboratory equipment, thereby raising investment and maintenance expenses. The recovery of KOH after activation is also difficult and economically unfeasible, since the reaction products, such as K_2_CO_3_, undergo partially irreversible transformations, making the activator suitable only for single use. Moreover, in cases of improper waste neutralization, potassium-containing solutions may pose an environmental hazard, leading to water and soil contamination, which requires the implementation of costly and energy-intensive purification procedures.

In comparison with KOH, the use of K_2_CO_3_ as an activating agent offers several significant advantages that make the activation process safer, cleaner, and more sustainable [17]. The amount of alkaline wastewater generated after activation is considerably lower, and the post-treatment solutions are easier to neutralize, since K_2_CO_3_ produces fewer soluble inorganic residues than KOH. Moreover, K_2_CO_3_ is a mild, non-corrosive, and stable compound, which greatly reduces equipment degradation and improves the overall safety of the process. Its non-toxic and environmentally benign nature minimizes ecological risks in case of accidental release, in contrast to the highly caustic behavior of KOH. Another important benefit is the ease of handling and storage, as potassium carbonate is chemically stable and non-hygroscopic, allowing for simple and safe operation on both laboratory and industrial scales. Additionally, K_2_CO_3_ can be obtained from recycled streams or industrial residues containing potassium salts, which aligns well with the principles of green chemistry and circular economy, making it an attractive and eco-friendly alternative to conventional activating agents [20].

It is commonly believed that KOH is a superior activating agent for the production of activated carbons due to the higher CO_2_ adsorption capacity typically observed for such materials [21,22,23]. However, this is not always the case, as can be seen from the analysis of Table 1.

Therefore, we decided to investigate the activation of avocado seeds using K_2_CO_3_ instead of KOH, which had previously yielded high CO_2_ adsorption [24]. We were the first to describe the use of avocado seeds as a precursor for activated carbon for CO_2_ adsorption [24]. In that study, KOH was used as the activating agent. In the present manuscript, we demonstrate how avocado seeds can be efficiently converted into highly microporous CO_2_ sorbents using K_2_CO_3_—a non-corrosive, non-toxic, and environmentally friendly activating agent that offers a practical alternative to KOH. Previous studies involving avocado seeds relied mainly on KOH activation which, although effective, has significant drawbacks: (i) it generates large amounts of highly corrosive wastewater, (ii) it causes severe equipment corrosion, and (iii) it does not allow recovery of the activating agent. In contrast, our work shows for the first time that K_2_CO_3_ can replace KOH while still providing very high CO_2_ adsorption performance (up to 6.26 mmol/g at 0 °C and 1 bar).

Moreover, our study provides new insights by demonstrating that ultramicropores (0.3–0.7 nm) are formed most effectively when using a saturated K_2_CO_3_ solution, a 1:1 mass ratio of avocado seeds to K_2_CO_3_, and an activation temperature of 800 °C. These ultramicropores directly determine the highest CO_2_ adsorption.

Our work advances existing research by introducing a sustainable activation method that enables the production of highly efficient CO_2_ sorbents from avocado seeds, without the environmental and operational drawbacks associated with KOH.

This demonstrates that although KOH may in some cases achieve slightly higher values (as in our earlier work where 6.47 mmol/g was obtained), K_2_CO_3_ provides a very competitive performance considering its much lower environmental and operational cost.

## 2. Results and Discussion

**Figure 1:**
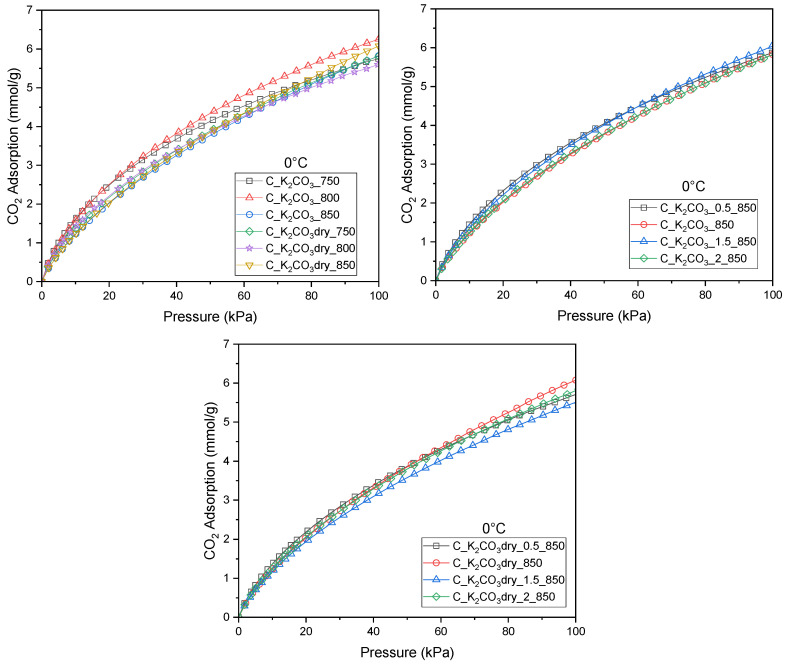
Isotherms of CO_2_ adsorption measured at 0 °C for activated biocarbons obtained from avocado seeds using K_2_CO_3_ as the activating agent.

Figure 1 presents CO_2_ adsorption at 0 °C on all activated biochars prepared using K_2_CO_3_ applied either in aqueous or dry form. In Figure 1, the materials activated at different temperatures with dry K_2_CO_3_ and with its saturated aqueous solution are grouped together for comparison. Figure 1 show the materials obtained at various mass ratios of the carbon precursor to the activating agent, using K_2_CO_3_ in solution (Figure 1) and in dry form (Figure 1).

The highest CO_2_ adsorption capacity (6.26 mmol/g) (Table 2) was obtained for the activated carbon produced using a saturated solution of the activating agent, a mass ratio of dried seeds to K_2_CO_3_ of 1:1, and an activation temperature of 800 °C. Deviations from the 1:1 ratio, either increasing or decreasing the amount of activating agent, resulted in only minor variations in CO_2_ adsorption and, in every case, led to a decrease in adsorption capacity, regardless of whether the activator was used in solid or solution form. Therefore further analyses focused exclusively on the activated carbons prepared with a 1:1 precursor-to-activating-agent ratio.

In [24], the activation of avocado seeds with KOH led to the formation of materials with very high specific surface areas (up to approximately 2000 m^2^/g), dominated by wider micropores and some mesopores, resulting from the intensive chemical etching characteristic of strongly alkaline activators. In contrast, activation with K_2_CO_3_ leads to a higher proportion of ultramicropores (0.3–0.7 nm), which are known to be crucial for CO_2_ adsorption at low temperatures; a lower contribution of mesopores, which enhances the adsorption potential within narrow pores; and a milder, more controllable activation mechanism (without strong corrosion), allowing for more precise tailoring of the microporous structure. As a result, although the specific surface area obtained with K_2_CO_3_ is lower than in the KOH-activated material, the proportion of ultramicropores is higher, which compensates for differences in SBET and explains the high CO_2_ uptake. A comparison of CO_2_ adsorption performance (K_2_CO_3_ vs. KOH) at 0 °C and 100 kPa shows that KOH activation yields 6.47 mmol/g (0 °C, 1 bar), whereas K_2_CO_3_ yields 6.26 mmol/g. This difference is small, and considering the milder nature of K_2_CO_3_, the absence of corrosive wastewater, the lack of equipment corrosion, the scalability, and the overall process safety, the achieved CO_2_ adsorption capacity is fully comparable while being more environmentally sustainable.

In the case of K_2_CO_3_, the activation mechanism at elevated temperatures (≥700 °C) can be summarized by the following reactions:

(1) K_2_CO_3_(s) → K_2_O(s) + CO_2_(g)

(2) K_2_CO_3_(s) + 2C(s) → 2K(g,l) + 3CO(g)

(3) K_2_O(s) + C(s) → 2K(g,l) + CO(g)

(4) C(s) + CO_2_(g) → 2CO(g)

In our system, these reactions lead to the in situ formation of CO and CO_2_, which gasify the carbon matrix and generate both micro- and macroporous structures. At the same time, the metallic potassium formed (via reactions (2) and (3)) can intercalate between carbon layers, causing their expansion; its removal during washing leaves behind a well-developed pore network. The release of CO and CO_2_ within the carbon matrix is therefore responsible for the development of both micropores and wider pores.

KOH also decomposes to K_2_CO_3_ during activation. However, the overall activation chemistry of KOH is more complex and significantly more aggressive, as it involves additional steps such as: 

(5) 2KOH(s) → K_2_O(s) + H_2_O(g)

(6) K_2_O(s) + CO_2_(g) → K_2_CO_3_(s)

and reactions leading to hydrogen evolution at high temperatures, for example:

(7) 2KOH(s) + C(s) → K_2_CO_3_(s) + H_2_(g)

(8) C(s) + H_2_O(g) → CO(g) + H_2_(g)

These reactions generate substantial amounts of H_2_O and H_2_, in addition to CO and CO_2_, which results in significantly stronger chemical etching.

The CO_2_ adsorption capacity of our best-performing sample (C_K_2_CO_3__800), equal to 6.26 mmol/g at 0 °C and 1 bar, compares very favorably with activated carbons reported in the literature, as summarized in Table 1. For KOH-activated carbons, the majority of materials exhibit CO_2_ uptakes in the range of 5.0–6.5 mmol/g: petroleum coke (6.08 mmol/g), waste tea (2.32 mmol/g). For K_2_CO_3_-activated carbons, reported capacities typically fall within 4.9–6.1 mmol/g: bamboo-derived carbon (6.08 mmol/g), sugarcane (4.90 mmol/g). Against this background, the capacity of our material (6.26 mmol/g) is among the highest reported for K_2_CO_3_-activated carbons, and is comparable to or higher than many KOH-activated carbons, despite the milder, non-corrosive nature of K_2_CO_3_. This clearly demonstrates that avocado-seed-derived activated carbons prepared using K_2_CO_3_ not only match, but also in several cases surpass the performance of conventional activated carbons, highlighting their strong potential for CO_2_ capture applications.

**Figure 2:**
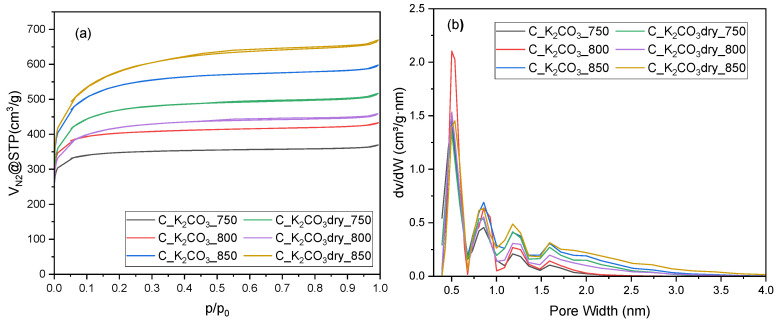
Nitrogen sorption isotherms (**a**) and pore size distribution profile (**b**) for activated biocarbons obtained from avocado seeds using K_2_CO_3_ as the activating agent.

Nitrogen adsorption–desorption isotherms at 196 °C for activated biocarbons prepared using K_2_CO_3_ as the activating agent, applied either as a saturated aqueous solution (C_K_2_CO_3_) or as a dry salt (C_K_2_CO_3_dry), at activation temperatures of 750, 800, and 850 °C are shown in Figure 2a. All samples exhibit type I isotherms with a pronounced nitrogen uptake at low relative pressures (p/p_0_ < 0.1), characteristic of a microporous structure. A slight increase in adsorption at higher relative pressures and the absence of a noticeable hysteresis loop suggest the presence of a small amount of mesopores. The highest nitrogen adsorption was observed for the sample activated with dry K_2_CO_3_ at 850 °C, indicating the largest pore volume. The material obtained at the same temperature using the K_2_CO_3_ solution showed a lower nitrogen uptake (the second highest among the samples). The activated carbon that exhibited the highest CO_2_ adsorption capacity (C_K_2_CO_3__800) showed a relatively low nitrogen adsorption. The sample exhibited the second-lowest nitrogen adsorption volume at relative pressure near unity, indicating limited pore development compared with the other carbons.

The pore size distribution curves (Figure 2b), obtained from the N_2_ adsorption data using the DFT model, confirm the predominance of micropores in all samples, with a main peak centered around 0.5 nm. A minor contribution of wider micropores and narrow mesopores (1–2 nm) is also visible, especially for the carbons activated at higher temperatures. The C_K_2_CO_3__800 material shows the highest and most intense peak at 0.5 nm, indicating the greatest proportion of ultramicropores, which explains its excellent CO_2_ adsorption performance. In contrast, the carbons activated at 850 °C, particularly those obtained using dry K_2_CO_3_, exhibit broader pore distributions extending toward the mesopore range, suggesting progressive pore widening with increasing activation temperature. The obtained results are consistent with the shape of the N_2_ adsorption isotherms (Figure 2a), confirming that activation with dry K_2_CO_3_ promotes a more intensive development of the total porosity compared with activation using the aqueous solution.

The preliminary analysis of Figure 2 indicates that high total pore volume does not necessarily determine high CO_2_ adsorption capacity. Table 3 presents the textural properties of the activated biocarbons, namely the specific surface area, total pore volume, and micropore volume. The values of these parameters are consistent with the adsorption isotherm profiles and confirm the conclusions drawn from Figure 2. A more detailed analysis of the textural parameters, aimed at identifying possible correlations between each of them and the CO_2_ adsorption capacity, revealed that their values do not directly influence CO_2_ uptake (Figure S1).

The exceptionally high surface area of 2076 m^2^/g results from a synergistic effect of two factors: the use of dry K_2_CO_3_ and a high activation temperature of 850 °C. Individually, neither variable explains the result: For the dry-activated samples, the BET surface area does not increase monotonically with temperature (1738 m^2^/g at 750 °C, decreasing to 1562 m^2^/g at 800 °C, and increasing again to 2076 m^2^/g at 850 °C). For the solution-activated samples, the highest surface areas remain significantly lower (1365–1951 m^2^/g). This indicates that only the combination of dry K_2_CO_3_ with the highest activation temperature (850 °C) provides conditions that favor extensive CO_2_—carbon gasification and the generation of both micropores and wider pores—resulting in the maximum surface area observed. Therefore, we conclude that: The dominant factor is the synergistic action of high activation temperature (850 °C) and the dry form of K_2_CO_3_, rather than either variable alone.

An attempt was also made to identify a correlation between the cumulative pore volume restricted to pores of a specific size range. The highest correlation coefficient was obtained for pores not larger than 1.08 nm, reaching a value of 0.63. This indicates a moderate positive correlation between the analyzed variables. Considering Figure 2b, it can be assumed that CO_2_ adsorption is primarily influenced by pores in the range of 0.3–0.7 nm, although pores with sizes between 0.7 and 1 nm also have a certain contribution.

**Figure 3:**
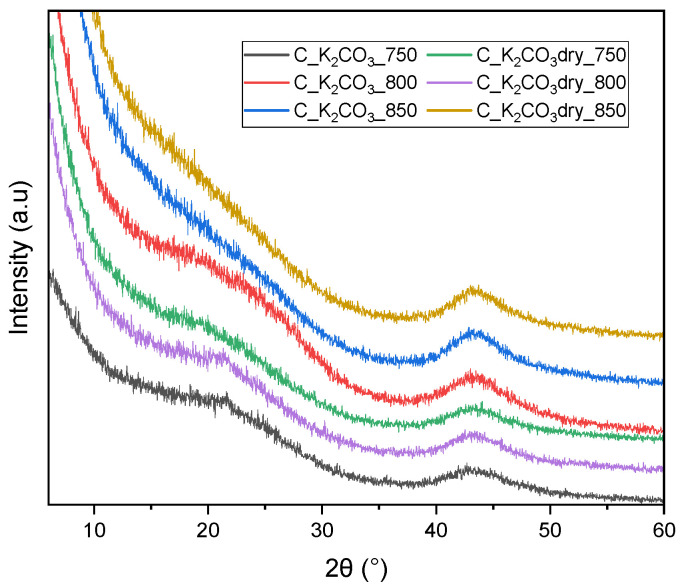
X-ray diffraction patterns of activated biocarbons obtained from avocado seeds using K_2_CO_3_ as the activating agent.

The XRD patterns (Figure 3) of all activated biocarbons obtained using K_2_CO_3_ as the activating agent display a broad diffraction peak centered at approximately 2θ ≈ 43°, corresponding to the (100) plane of turbostratic carbon. This indicates a certain degree of ordering of carbon atoms within the sp^2^ structure. Only for the samples C_K_2_CO_3__750, C_K_2_CO_3__800, and C_K_2_CO_3_dry_800, a very weak peak at around 2θ ≈ 23°, corresponding to the (002) plane, can be observed. This suggests that well-ordered graphene layers are absent in the carbon structure of all materials, with only a very low degree of ordering in the mentioned samples. No crystalline potassium-containing phases were detected, confirming efficient washing and removal of inorganic residues after activation. Based on the XRD results and CO_2_ adsorption data, it is evident that amorphous/turbostratic carbon is more favorable for CO_2_ capture than graphitized carbon. The samples exhibiting the most disordered structures (broad (100) peak and negligible (002) reflection) developed the highest ultramicropore volume and consequently showed the highest CO_2_ uptake.

In addition to the CO_2_ adsorption measurements at 0 °C, experiments were also carried out at 10 °C and 30 °C to obtain a more comprehensive description of the adsorption equilibrium and its temperature dependence, enabling a more detailed analysis and mathematical modeling.

**Figure 4:**
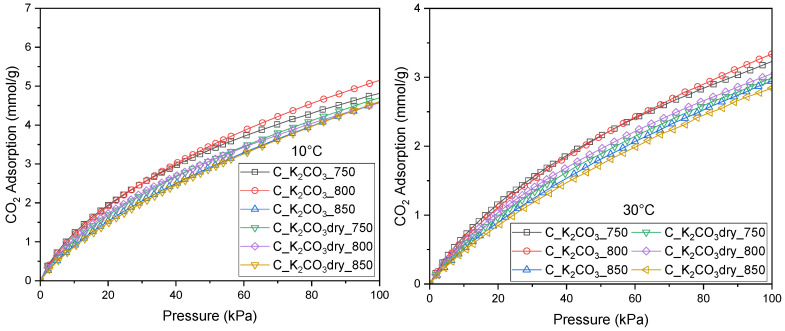
Isotherms of CO_2_ adsorption measured at 10 °C and 30 °C for activated biocarbons obtained from avocado seeds using K_2_CO_3_ as the activating agent.

CO_2_ adsorption isotherms obtained at 0, 10, and 30 °C (Figure 4) were analyzed using the Langmuir, Freundlich, Sips, Toth, and Radke–Prausnitz models [37]. The HYBRID error function (1) was employed to identify the best-fitting adsorption isotherm model.

(1) $$HYBRID=\frac{100}{n-p}\sum_{i=1}^{n}\frac{{\left(q_{i,exp}-q_{i,calc}\right)}^{2}}{q_{i,exp}}$$

q_e,calc_—theoretical value of adsorption on the activated carbon surface calculated based on the model;

q_e,exp_—experimental value of adsorption on the activated carbon surface;

n—total number of measurements;

p—number of model parameters.

Nonlinear regression was performed in Excel 365 employing the Solver add-in to identify the model that provided the best fit to the experimental data. The Sips equation is defined by (2):

(2) $$q=\frac{q_{m}\cdot b\cdot p^{n}}{1+b\cdot p^{n}}$$

where

q—the CO_2_ equilibrium adsorption at p;

p—equilibrium pressure;

q_m_—the saturation capacity;

b—equilibrium constant;

n—exponential parameter indicating the heterogeneity of the sorbent.

The optimized parameters of the Sips equation together with the corresponding HYBRID values at 0, 10, and 30 °C are summarized in Table 4. The n values notably differ from unity, reflecting the heterogeneous nature of the surface of all investigated activated carbons.

**Figure 5:**
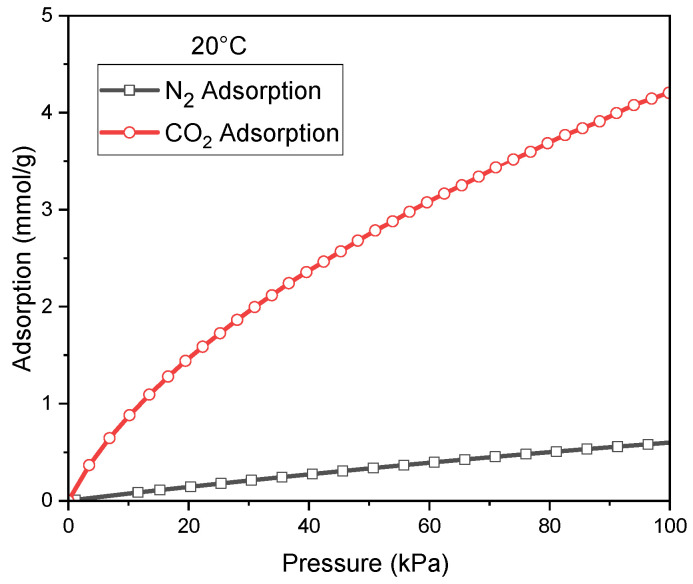
Comparison of N_2_ and CO_2_ adsorption at 20 °C for C_K_2_CO_3__800.

For the best CO_2_ adsorbent (C_K_2_CO_3__800), additional CO_2_ and N_2_ adsorption measurements were carried out at 20 °C Figure 5. Comparison of N_2_ and CO_2_ adsorption at 20 °C for C_K_2_CO_3__800. This enabled the construction of reliable temperature-dependent relationships for the parameters qₘ, b, and n, and for the CO_2_/N_2_ adsorption selectivity.

Mathematical analysis of the data presented in Figure 5 indicated that, for both CO_2_ and N_2_, the Sips model provided the best fit to the adsorption equilibrium. The calculated parameters are summarized in Table 5.

The parameters in the Sips Equation (2) are temperature dependent:

(3) $$q_{m}=q_{m0}\cdot exp\left[\chi \left(1-\frac{T}{T_{0}}\right)\right],$$

(4) $$b=b_{0}exp\left[\frac{Q}{RT}\left(\frac{T_{0}}{T}-1\right)\right],$$

(5) $$n=n_{0}+\alpha \left(1-\frac{T_{0}}{T}\right),$$

In Equations (3)–(5) q_mo_, χ, Q, b_0_, n_0_, and α are the constants. R is ideal gas constant. T_0_ is the reference temperature. In this work, the lowest temperature (273 K) was chosen as the reference temperature.

**Figure 6:**
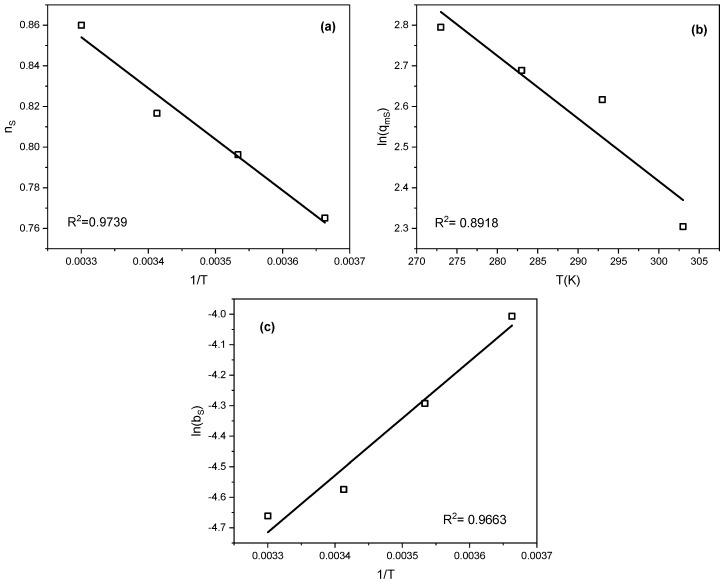
Dependence of (**a**) n_S_, (**b**) ln(q_mS_), and (**c**) ln(b_S_) on temperature and its inverse.

To determine the parameter q_m0_, a plot of ln(q_m_) versus temperature was constructed. The parameters Q and b_0_ were derived from the linear dependence of b on 1/T. Similarly, n_0_ and α were obtained from the n versus 1/T relationship. These correlations are illustrated in Figure 6. In all cases, linear relationships with high correlation coefficients were observed. This allowed the determination of additional parameters of the equations defining the temperature dependencies. The parameters are summarized in Table 6.

In addition to the high CO_2_ adsorption capacity, the selectivity of CO_2_ over nitrogen, the main component of flue gases, is also of great importance. The CO_2_/N_2_ adsorption selectivity was evaluated using the Ideal Adsorbed Solution Theory (IAST) [38].Within this theoretical framework, the selectivity of component A over component B in a binary gas mixture can be derived from the individual adsorption isotherms of both components. The selectivity equilibrium is expressed by Equation (6):

(6) $$S_{eq}=\frac{q_{{CO}_{2}(p)}}{q_{N_{2}(p)}},$$

**Figure 7:**
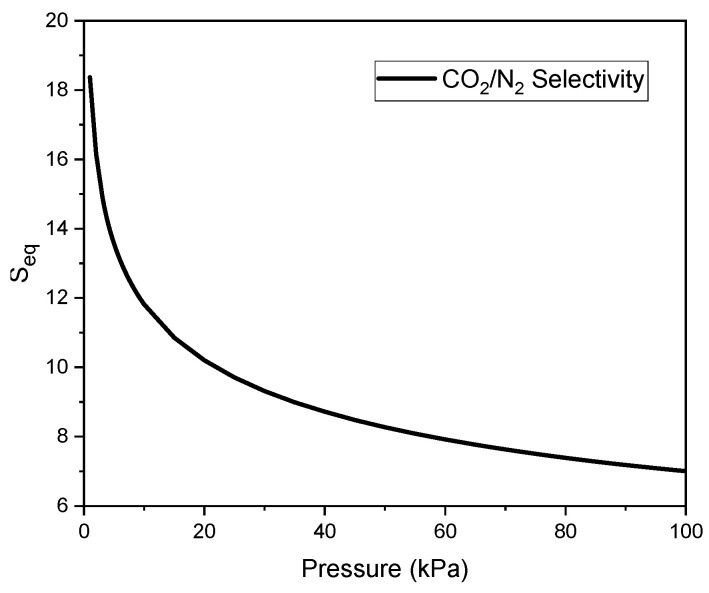
Equilibrium selectivity of C_K_2_CO_3__800 for CO_2_ relative to N_2_.

Using the parameter values presented in Table 6**,** the adsorption of CO_2_ ($q_{{CO}_{2}(p)}$) and N_2_ ($q_{N_{2}(p)}$) was calculated for the same pressure values. Figure 7 shows the calculated values of CO_2_/N_2_ adsorption selectivity for an equimolar mixture at 20 °C as a function of pressure.

The equilibrium selectivity at low pressures is the highest, reaching a value of 18. It then decreases to approximately 8 at a pressure of 40, while further pressure increase results in only minor changes.

Nitrogen constitutes the dominant component of flue gases, typically representing more than 70% of the dry volume. This results from the fact that air, used as the oxidizing medium in combustion, contains about 78% nitrogen. As nitrogen remains largely inert under standard combustion conditions, it passes through the process without significant chemical transformation.

Conversely, carbon dioxide is the principal product of the complete oxidation of organic carbon in fossil fuels and biomass. Its concentration in flue gases depends on the fuel type and oxygen availability, generally ranging from 10% to 20% [39].

For the evaluation of CO_2_/N_2_ adsorption selectivity, gas mixtures reflecting the typical composition of flue gases were considered, namely: 10% CO_2_ and 90% N_2_, 15% CO_2_ and 85% N_2_, and 20% CO_2_ and 80% N_2_. For this purpose, Equation (7) was applied

(7) $$S_{CO2\%}=\frac{q_{{CO}_{2}(p)}/q_{N_{2}(p)}}{p_{{CO}_{2}}/p_{N_{2}}},$$

where CO_2_% is the CO_2_ content in the CO_2_/N_2_ gas mixture.

The calculated CO_2_/N_2_ adsorption selectivity for C_K_2_CO_3__800 under model flue gas compositions at temperature of 20 °C are presented in Table 7.

The CO_2_ adsorption selectivity values for C_K_2_CO_3__800 under model flue gas compositions are high, which makes this material a promising CO_2_ sorbent for various types of flue gases.

The determination of the isosteric heat of adsorption (*Q*_iso_) is essential for understanding the energetics of the adsorption process. This parameter provides valuable insight into the strength and nature of the interaction between the adsorbate molecules and the surface of the adsorbent. High *Q*_iso_ values indicate strong adsorbate–adsorbent interactions, typically associated with microporous structures or the presence of specific active sites, while lower values reflect weaker physical adsorption. Moreover, *Q*_iso_ is crucial for evaluating the regeneration energy requirements of sorbents and for assessing their practical applicability in cyclic adsorption–desorption processes, such as CO_2_ capture.

The isosteric heat of adsorption was calculated using Clausius—Clapeyron Equation (8):

(8) $$Q_{iso}=-R{\left(\frac{\partial ln(p)}{\partial \left(\frac{1}{T}\right)}\right)}_{\theta },$$

where

R is the universal gas constant,

T is the temperature, p is the pressure,

θ is the degree of surface coverage.

After integrating Equation (8), a linear function is obtained (9):

(9) $${\ln\left(p\right)}_{\theta }=-\frac{Q_{iso}}{R}\frac{1}{T}+C$$

**Figure 8:**
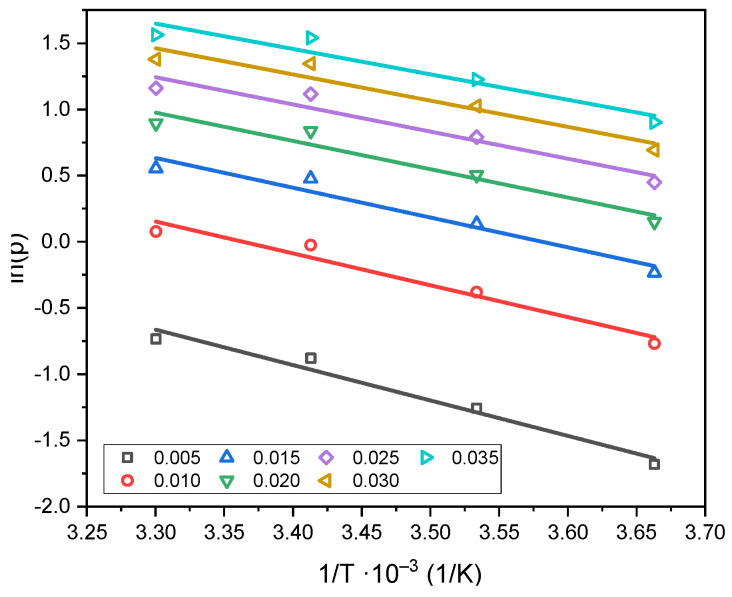
Adsorption isosteres corresponding to various degrees of surface coverage on C_K_2_CO_3__800.

Plots of the logarithm of equilibrium pressure versus the reciprocal of temperature were constructed for several fixed surface coverages (adsorption isosteres), as shown in Figure 8.

**Figure 9:**
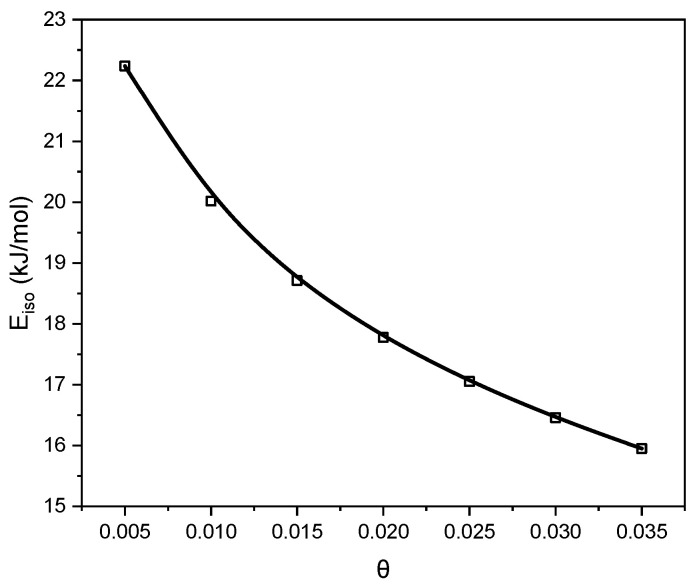
Variation in the isosteric heat of CO_2_ adsorption with surface coverage for C_K_2_CO_3__800, calculated from the isosteres presented in Figure 8.

The isosteric heat of adsorption (Figure 9) ranged from 22 kJ/mol for the low surface coverage. These values confirmed that CO_2_ adsorption on the C_K_2_CO_3__800 proceeds predominantly through a physical mechanism. A gradual decrease in the isosteric heat with increasing surface coverage reflects the weakening of interactions between CO_2_ molecules and the carbon surface. CO_2_ is bound mainly through van der Waals forces, which explains its relatively easy desorption from the adsorbent surface.

## 3. Materials and Methods

### 3.1. Materials

Analytical-grade reagents, including K_2_CO_3_—activating agent and hydrochloric acid (35–38%, Chempur, Łódź, Poland), were used. Activated biocarbons were synthesized using avocado seeds as the carbon precursor. The seeds were dried at 60 °C for 24 h and ground.

### 3.2. Synthesis of Activated Carbons

**Figure 10:**
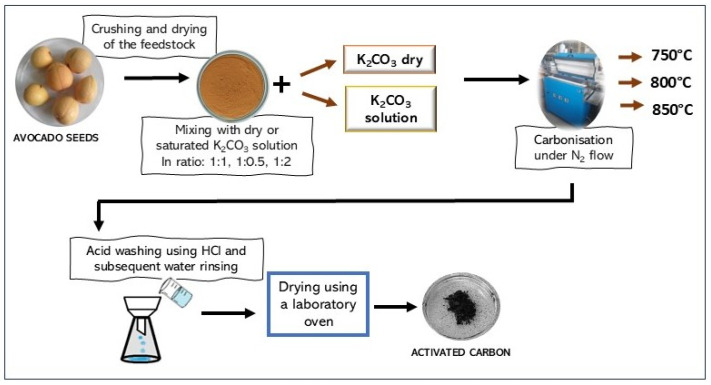
Preparation pathway of activated biocarbons obtained from avocado seeds using K_2_CO_3_ as the activating agent.

Twelve activated biocarbons were produced from avocado seed powder under various conditions, as summarized in Table 8 and illustrated schematically in Figure 10.

The dried and powdered avocado seeds were impregnated with either a saturated solution of K_2_CO_3_ or dry K_2_CO_3_. Different mass ratios of avocado seeds to the activating agent were applied (Table 8). The samples prepared using the K_2_CO_3_ solution were dried in a dryer at 190 °C prior to carbonization. Combined carbonization and chemical activation were carried out in a tubular furnace at various temperatures (Table 8) for 1 h under a continuous nitrogen flow. The resulting materials were subsequently washed with 1 M HCl and rinsed with distilled water until a neutral pH was reached.

### 3.3. Sample Characterization

The porous structure of the synthesized activated carbons was characterized by N_2_ adsorption–desorption measurements at −196 °C (ASAP 2460, Micromeritics, Norcross, GA, USA), from which the specific surface area (S_BET_), total pore volume (V_tot_), micropore volume (V_micro_), and pore size distribution were evaluated. The specific surface area (SBET) was determined using the Brunauer–Emmett–Teller (BET) model within the relative pressure range of 0.05–0.30, typically suitable for nonporous and mesoporous materials. Given that the investigated carbons were mainly microporous, the modified BET evaluation proposed by Rouquerol et al. [40] was employed. This approach ensures the physical relevance of the BET parameters by satisfying two conditions: (i) a positive BET constant (C > 0) and (ii) a monotonic increase in the V(1 − p/p_0_) function with p/p_0_. The total pore volume was assessed from the nitrogen adsorption capacity at relative pressures close to unity. Micropore volume and pore size distribution were subsequently derived through density functional theory (DFT) analysis of the N_2_ isotherms, assuming slit-like pore geometry.

Adsorption isotherms were collected volumetrically with an ASAP 2460 analyzer (Micromeritics, Norcross, GA, USA). CO_2_ adsorption was studied over the temperature range of 0–30 °C, while N_2_ adsorption was evaluated at 20 °C and pressures up to 1 bar.

X-ray diffraction (XRD) patterns of the activated carbons were collected on a PANalytical Empyrean diffractometer (X’Pert–PRO, Panalytical, Almelo, The Netherlands, 2012) operated with Cu Kα radiation to assess their crystalline structure.

## 4. Conclusions

Activated biocarbons were successfully synthesized from avocado seeds using potassium carbonate (K_2_CO_3_) as an activating agent. The results demonstrated that K_2_CO_3_ can serve as an efficient, environmentally friendly, and non-corrosive alternative to the traditionally used KOH, while still enabling the formation of highly microporous carbon materials with excellent adsorption performance. The sample C_K_2_CO_3__800, prepared using a saturated aqueous K_2_CO_3_ solution at an activation temperature of 800 °C and a 1:1 precursor-to-activating-agent ratio, exhibited the highest CO_2_ adsorption capacity of 6.26 mmol/g at 0 °C and 1 bar.

The CO_2_ adsorption capacity of 6.26 mmol/g obtained in our work is fully consistent with the expected performance of K_2_CO_3_-activated carbons derived from avocado seeds. Values above 6 mmol/g are generally considered high for this activation system. The material described in [19], which shows an exceptionally high uptake of 7.18 mmol/g, was produced from spent coffee grounds, a precursor with a substantially higher oxygen content and significantly greater reactivity toward K_2_CO_3_, which strongly promotes the development of a very large volume of ultramicropores (<0.5 nm). In contrast, avocado seeds contain more lignin and ash and therefore develop a less extreme ultramicroporous structure under identical activation conditions.

Thus, the difference in CO_2_ uptake arises primarily from the distinct precursor chemistry and the associated mechanisms of textural development. The value of 6.26 mmol/g reported in this work is high for avocado seed-based carbons activated with K_2_CO_3_ and lies within the upper range typically observed for this activation system.

Textural analysis revealed that the outstanding CO_2_ uptake of this material was associated with the predominance of ultramicropores (0.3–0.7 nm), which are particularly effective for CO_2_ molecule confinement. Although samples activated at higher temperatures showed a greater total pore volume, this did not directly translate into higher CO_2_ adsorption, confirming that pore size distribution—rather than surface area alone—plays a decisive role in determining sorption efficiency.

The adsorption equilibrium data were best described by the Sips model, confirming the heterogeneous nature of the surface of the investigated carbons. The CO_2_/N_2_ selectivity analysis based on the Ideal Adsorbed Solution Theory (IAST) indicated high separation performance, with equilibrium selectivity values up to 18 at low pressures and 11–14 under flue-gas-like compositions. The isosteric heat of adsorption, ranging from 22 to 26 kJ/mol, confirmed the predominance of physisorption, ensuring good regenerability of the sorbent.

Overall, the study confirms that avocado seed-derived activated carbons prepared with K_2_CO_3_ are promising candidates for CO_2_ capture applications. Their combination of high adsorption capacity, good selectivity, and sustainable synthesis route based on a renewable precursor highlights the potential of this approach for the development of efficient, low-cost, and environmentally responsible sorbent materials.

## Figures and Tables

**Table 1:** CO_2_ capture comparison of activated carbons from different feedstocks at 0 °C and 1 bar.

Feedstock	Activator	Adsorption	Reference
avocado seeds	KOH	6.47	[24]
petroleum coke	KOH	6.08	[25]
coal tar pitch	KOH	6.00	[26]
rice husk	KOH	5.70	[27]
pollens	KOH	5.63	[28]
nitrogen-free phenolic resin	KOH	5.07	[29]
argan paste cakes	KOH	4.58	[30]
waste tea	KOH	2.32	[31]
bamboo powder	K_2_CO_3_	6.08	[32]
bamboo shoot	K_2_CO_3_	5.93	[33]
cotton waste	K_2_CO_3_	5.37	[34]
peanut shell	K_2_CO_3_	5.21	[35]
coconut shell	K_2_CO_3_	5.12	[17]
sugarcane	K_2_CO_3_	4.90	[36]

**Table 2:** Carbon dioxide adsorption at 0 °C and 100 kPa for activated biocarbons obtained from avocado seeds using K_2_CO_3_ as the activating agent.

Sample Name	CO_2_ Adsorption (mmol/g)
C_K_2_CO_3__750	5.75
C_K_2_CO_3__800	6.26
C_K_2_CO_3__850	5.83
C_K_2_CO_3_dry_750	5.81
C_K_2_CO_3_dry_800	5.60
C_K_2_CO_3_dry_850	6.08
C_K_2_CO_3__0.5_850	5.87
C_K_2_CO_3__1.5_850	6.03
C_K_2_CO_3__2_850	5.85
C_K_2_CO_3_dry_0.5_850	5.73
C_K_2_CO_3_dry_1.5_850	5.54
C_K_2_CO_3_dry_2_850	5.84

**Table 3:** Textural properties of activated biocarbons obtained from avocado seeds using K_2_CO_3_ as the activating agent.

Activated Carbon	BET (m^2^/g)	Vtot (cm^3^/g)	Vmicro (cm^3^/g)
C_K_2_CO_3__750	1365	0.572	0.460
C_K_2_CO_3__800	1573	0.534	0.670
C_K_2_CO_3__850	1951	0.925	0.658
C_K_2_CO_3_dry_750	1738	0.800	0.585
C_K_2_CO_3_dry_800	1562	0.710	0.528
C_K_2_CO_3_dry_850	2076	1.036	0.670

**Table 4:** Parameters of the Sips equation at 0 °C, 10 °C, and 30 °C and the corresponding HYBRID values for CO_2_ adsorption on activated biocarbons obtained from avocado seeds using K_2_CO_3_ as the activating agent.

	C_K_2_CO_3__750	C_K_2_CO_3__800	C_K_2_CO_3__850	C_K_2_CO_3_dry_750	C_K_2_CO_3_dry_800	C_K_2_CO_3_dry_850
0 °C						
q_m_	13.012	16.358	22.012	20.601	16.640	28.778
b	0.025	0.018	0.009	0.012	0.017	0.007
n	0.750	0.765	0.791	0.748	0.742	0.780
HYBRID	0.0072	0.0065	0.0052	0.0078	0.0046	0.0086
10 °C						
q_m_	11.490	14.711	17.455	16.408	13.454	20.184
b	0.020	0.014	0.008	0.011	0.014	0.007
n	0.781	0.796	0.815	0.782	0.777	0.818
HYBRID	0.0052	0.0037	0.0061	0.0048	0.0063	0.0043
30 °C						
q_m_	7.782	10.017	11.243	9.387	8.876	11.654
b	0.014	0.009	0.006	0.009	0.010	0.006
n	0.854	0.860	0.876	0.855	0.845	0.882
HYBRID	0.0018	0.0038	0.0037	0.0024	0.0041	0.0033

**Table 5:** The parameters of the Sips equation at 20 °C and the corresponding HYBRID values for CO_2_ and N_2_ adsorption for C_K_2_CO_3__800.

C_K_2_CO_3__800 20 °C	CO_2_	N_2_
q_m_	13.693	3.203
b	0.010	0.002
n	0.817	0.993
HYBRID	0.0040	0.0002

**Table 6:** Parameters of the Sips Equations (3)–(5) describing temperature-dependent adsorption behavior.

Parameter	Values	Unit
Q	15,527	J/mol
b_0_	0.0099	bar^−1^
n_0_	0.763	
α	0.931	
q_m0_	16.99	mmol/g
χ	4.212	

**Table 7:** Calculated CO_2_ over N_2_ adsorption selectivity for C_K_2_CO_3__800 under model flue gas.

Flue Gas [CO_2_/N_2_]	S_eq_
10/90	14.15
15/85	12.69
20/80	11.65

**Table 8:** Preparation conditions and sample designations of activated biocarbons obtained from avocado seeds using K_2_CO_3_ as the activating agent.

Sample Name	Form of Activating Agent	Carbon Source to Activating Agent Ratio	Carbonization/Activation Temperature
C_K_2_CO_3__750	K_2_CO_3_ solution	1:1	750
C_K_2_CO_3__800	K_2_CO_3_ solution	1:1	800
C_K_2_CO_3__850	K_2_CO_3_ solution	1:1	850
C_K_2_CO_3_dry_750	K_2_CO_3_ dry	1:1	750
C_K_2_CO_3_dry_800	K_2_CO_3_ dry	1:1	800
C_K_2_CO_3_dry_850	K_2_CO_3_ dry	1:1	850
C_K_2_CO_3__0.5_850	K_2_CO_3_ solution	1:0.5	850
C_K_2_CO_3__1.5_850	K_2_CO_3_ solution	1:1.5	850
C_K_2_CO_3__2_850	K_2_CO_3_ solution	1:2	850
C_K_2_CO_3_dry_0.5_850	K_2_CO_3_ dry	1:0.5	850
C_K_2_CO_3_dry_1.5_850	K_2_CO_3_ dry	1:1.5	850
C_K_2_CO_3_dry_2_850	K_2_CO_3_ dry	1:2	850

## Data Availability

Dataset available on request from the authors.

## Supplementary Materials

The following supporting information can be downloaded at: https://www.mdpi.com/article/10.3390/molecules30234658/s1, Figure S1. Attempts to find correlations between CO_2_ adsorption and specific surface area, total pore volume, and micropore volume.
