# Supplementary material for: Synthesis and Characterization of Activated Biocarbons Produced from Avocado Seeds Using the Non-Toxic and Environmentally Friendly Activating Agent K2CO3 for CO2 Capture
Source: Molecules. 2025 Dec 4;30(23):4658. doi: 10.3390/molecules30234658 (PMC12693009; doi:10.3390/molecules30234658)
Supplement: Supplementary file 1 [file molecules-30-04658-s001.zip › molecules-4010830-supplementary.pdf]

Synthesis and Characterization of Activated Biocarbons Produced from Avocado Seeds Using the Non-Toxic and Environmentally Friendly Activating Agent K_2CO_3 for CO_2 Capture

Joanna Siemak and Beata Michalkiewicz *

Department of Catalytic and Sorbent Materials Engineering, Faculty of Chemical Technology and Engineering, West Pomeranian University of Technology in Szczecin, Piastów Ave. 42, 71-065 Szczecin, Poland; joanna.siemak@zut.edu.pl

* Correspondence: beata.michalkiewicz@zut.edu.pl; Tel.: +48-91-449-4096; Fax: +48-91-449-4247

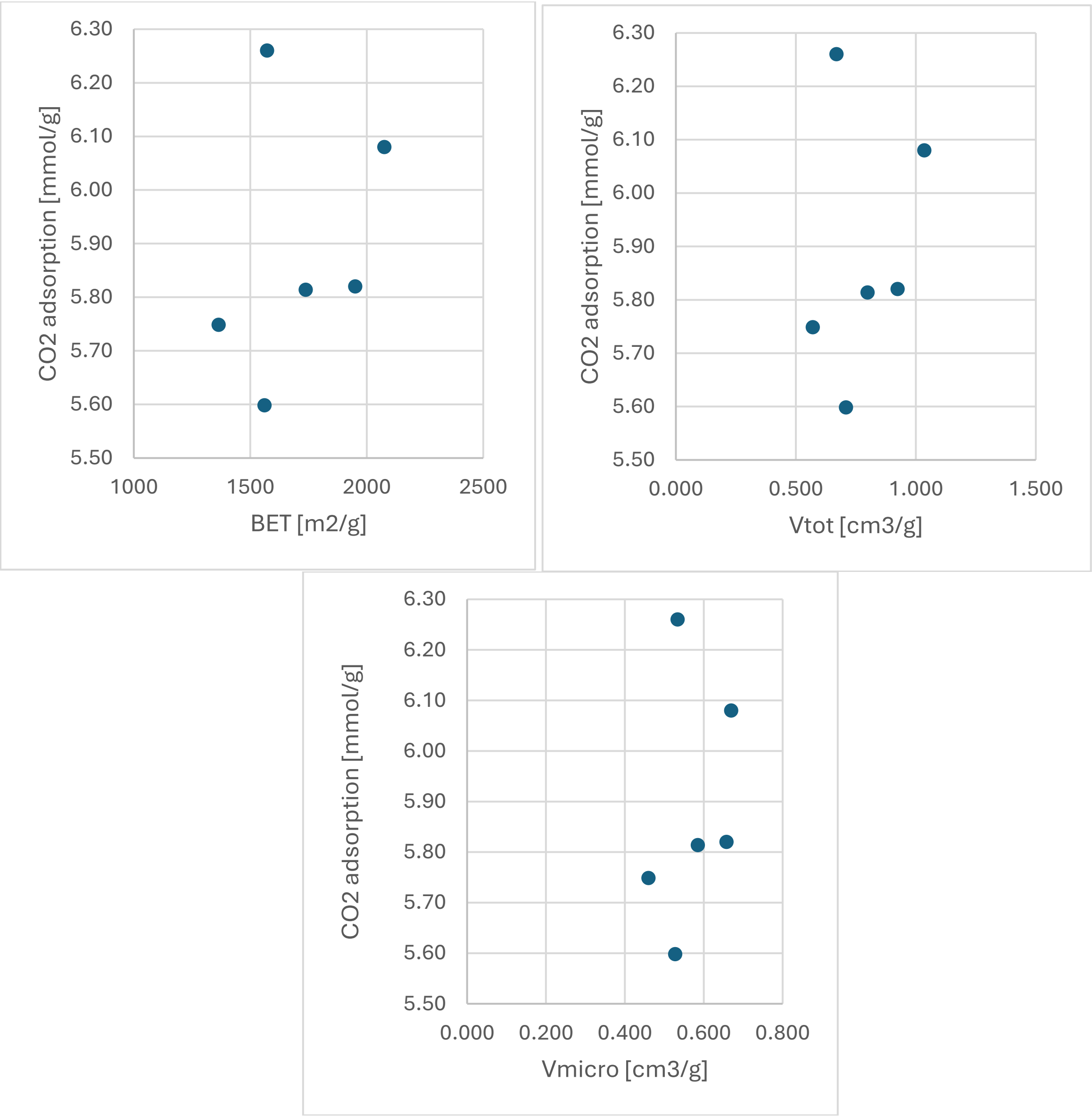

Figure S1. Attempts to find correlations between CO_2 adsorption and specific surface area, total pore volume, and micropore volume.
